# Electric Fields and Waves in the Venus Nightside Magnetosphere

**DOI:** 10.1007/s11214-025-01242-x

**Published:** 2025-11-11

**Authors:** F. S. Mozer, A. V. Agapitov, S. D. Bale, J. W. Bonnell, M. Pulupa, T. Quinn, A. Voshchepynets

**Affiliations:** 1 Space Sciences Laboratory, University of California, Berkeley, CA USA; 2 Physics Department, University of California, Berkeley, CA USA; 3 Uzhhorod National University, Uzhhorod, Ukraine

**Keywords:** Magnetospheres, Parker Solar Probe, Venus, Solar wind interaction, Electric fields measurements

## Abstract

On November 6, 2024, the Parker Solar Probe flew past Venus to make the first accurate electric field measurement in the nightside Venusian magnetosphere. To achieve this result, the electric field antennas were current biased in a way never before experienced by an electric field detector at Venus. This biasing requirement, that the positive bias current in the Venus shadow be about equal to the electron thermal current, is discussed and illustrated. About one minute of useful electric field data in the eight minute nightside magnetosphere crossing was obtained, during which the only feature observed was a few Hz signal. This result, along with the magnetic field measurements, showed that there were few if any electromagnetic waves, such as low frequency electromagnetic turbulence or whistlers, in the nightside crossing. Instead, a few Hertz, purely electrostatic signal was found. This suggests that the interaction of the solar wind with an unmagnetized body having an ionosphere may be different from that of previously studied magnetized bodies. In the sunlit flanks, many electromagnetic wave modes were observed. An additional result of this research is development of an improved algorithm for biasing electric field antennas in the Sun’s shadow which improves on guessing the bias current as done in this research.

## Plain Language Summary

The electric field instrument on the Parker Solar Probe had a bias current that was varied while the spacecraft was in the Venusian nightside magnetosphere, such that there were times when the bias current was appropriate for an accurate measurement of the electric field. Low frequency electrostatic turbulence was observed and no electromagnetic waves, such as whistlers, were seen.

## Introduction

The planet Venus does not have an intrinsic dipole-like magnetic field (Bridge et al. 1967). In the absence of such a field, the electrodynamic interaction between the Venus ionospheric plasma and the solar wind creates an induced magnetosphere that may drive a variety of plasma waves. Waves in magnetized plasmas, such as a turbulent cascade, MHD waves, whistler waves, etc., may not exist at Venus, in which case Venus has a plasma environment that has not been well-studied.

Electric field instruments have been flown near Venus on the Pioneer Venus Orbiter (Taylor et al. 1979; Scarf et al. 1980), Galileo (Gurnett et al. 1991), and Cassini (Gurnett et al. 2001) with a primary aim of determining whether lightning and associated whistler waves exist at Venus. Some evidence of such waves was presented while later analyses suggested that the spikes were largely produced by noise, not waves (Taylor et al. 1987). A more complete discussion of the purported evidence of lightning and whistlers at Venus is given elsewhere (Williams et al. 1983; Russell 1991; Lorenz 2018). In spite of all such discussions, it is the case that the electric field instruments on these earlier satellites were not capable of fully measuring dc and low frequency electric fields because they operated in a mode that made them more sensitive to current fluctuations than to dc and low frequency electric fields. The reason that the electric field instruments were not in useful operating modes is that a working low frequency electric field instrument requires application of a bias current to the antennas in order that their net current from photoemission, electron thermal flux, proton thermal flux, and other minor current sources, be close to zero, as is discussed in more detail in the following section.

**Fig. 1:**
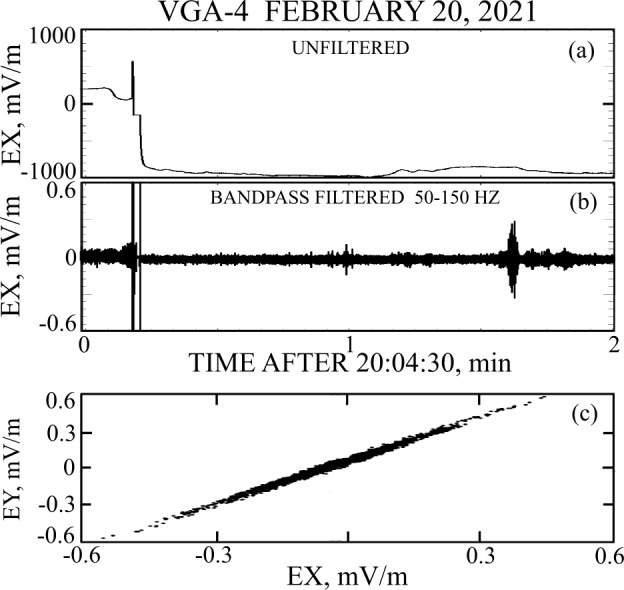
The electric field response when the spacecraft entered shadow. Panel (1a) shows that the field decreased from a reasonable value to the unphysical −1000 mV/m on entering the shadow. Panel (1b) provides bandpass filtered data indicating a small signal in shadow that is noise, and not a whistler wave, because the detector was near saturation. Panel (1c) confirms this result because the wave is not circular polarized, as would be required for a whistler

The Parker Solar Probe (PSP) flew biased electric field detectors with bias currents set for proper operation in sunlight. To provide Venus gravity assists, PSP had seven near-Venus flybys, the first six of which had this near constant bias current. Four of these flybys (VGA1, VGA2, VGA5, VGA6) encountered the foreshock, bow shock, or sunlit flanks, but not the nightside magnetosphere, during which good electric field measurements were made (George et al. 2024). Two of the first six flybys (VGA3 and VGA4) entered the nightside magnetosphere where, because there was no sunlight, the current biasing of the antennas was incorrect, and proper electric field measurements were not made. An example of this situation occurred on VGA4 (George et al. 2023), as illustrated in Fig. 1. In panel (1a), the spacecraft entered the nightside Venusian magnetosphere at about 20:04:40 when the electric field value changed from near zero to the unphysical −1000 mV/m, due to the current imbalance from the loss of photoemission. The 50-150 Hz bandpass filtered electric field in panel (1b) illustrates the purported whistler wave that occurred at about 20:06:10. Because the detector was more sensitive to current fluctuations than external potentials at this time, this observation is more likely current noise and not a whistler wave. This conclusion is strengthed in panel (1c) which provides the hodogram of the filtered EX versus EY and that shows the structure was linearly polarized and not at least partially circular polarized as required of a whistler wave. This example illustrates the extreme caution required when associating signals from improperly biased electric field sensors with physical phenomena.

VGA-7, the last Venus flyby, occurred on November 6, 2024, and had a bias current in the Venus shadow that allowed the nightside magnetospheric electric field at Venus to be measured for the first time. The relevant data for this measurement was provided by the FIELDS instrument (Bale et al. 2016). The biasing and the resulting fields will be considered following discussion of the algorithm for biasing the electric field sensors.

## Biasing the Electric Field Sensors

**Fig. 2:**
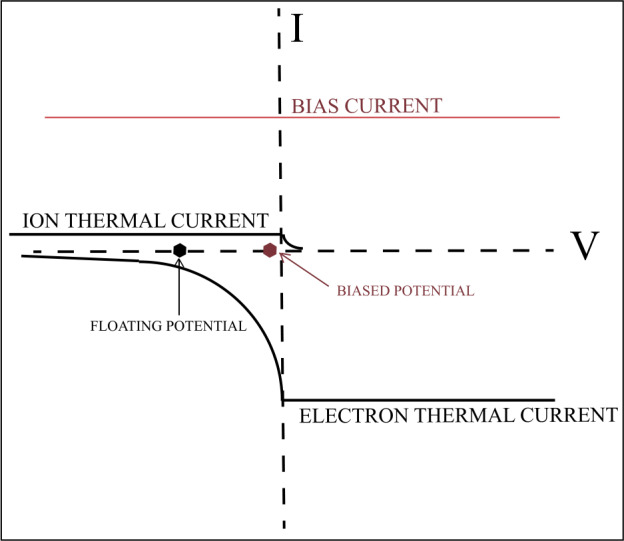
Schematic of Langmuir probe theory showing the location of the floating potential of a body experiencing only the proton and electron thermal currents and the biased potential of a body that experiences the additional bias current

The discussion of biasing electric field sensors requires consideration of simplified Langmuir probe theory. This theory gives the potential of a body with respect to the nearby plasma by requiring that, in equilibrium, the sum of all currents to the body be zero. Figure 2 provides a graphic illustration of this analysis with the potential of the body relative to the nearby plasma described along the X-axis and the current to the body given by the Y-axis. Suppose that the potential of the body is positive. Then the entire electron thermal current is attracted to become a negative current to the body, as illustrated in the figure (neglecting focusing effects, secondary emission, etc., for simplicity). When the body is negative, the electron thermal flux with energies less than the potential of the body is reflected, so the electron thermal current reaching the body decreases as the potential of the body becomes more negative, with the current decreasing exponentially with a scale equal to the electron temperature in a simple model. With opposite signs, the same phenomena happen to the proton thermal flux, with the proton current about a factor of 40 less than the electron thermal current because of the heavier proton mass. In the absence of photoemission or bias current (and neglecting other, smaller currents) the voltage at which the two currents are equal in magnitude and opposite in sign is at the illustrated negative voltage location called the floating potential. At this location, a small change of current produces a large change of the floating potential, as can be seen in Fig. 2, so the detector output is more dependent on current than voltage fluctuations. To resolve this problem, the potential of the body with respect to the nearby plasma should be nearly zero because, at this voltage, a change of current produces a much smaller change of the potential of the body. Thus, a positive bias current, such as that illustrated in the figure, makes the sum of all the currents equal to zero at the location labelled in the figure as the biased potential.

Applying the above discussion to an electric field detector, the spacecraft is at the floating potential and each antenna should be biased to be at or near the biased potential. The measured quantity, called the spacecraft potential, is then the difference of the biased potential and the floating potential which, for proper operation of the detector, should be a few volts positive in the absence of sunlight, as seen in the figure.

## Data

**Fig. 3:**
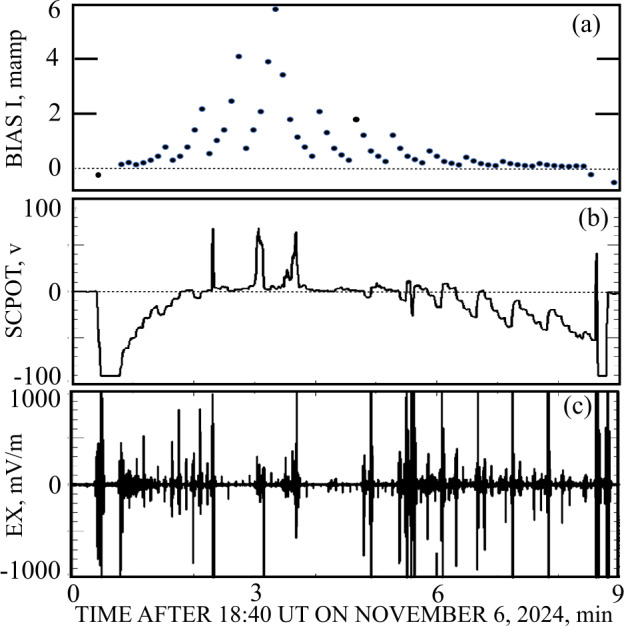
Panel (3a) illustrates the bias current applied to the antennas every seven seconds in an attempt to make the spacecraft potential of panel (3b) a few volts positive. Panel (3c) gives the electric field measured and that had a spike every seven seconds due to the changing bias current and that was large when the spacecraft potential differed from a small positive value

During VGA-7 on November 6, 2024, the spacecraft passed through the nightside Venusion magnetosphere for about eight minutes at an altitude as low as 400 kilometers. In the absence of photoemission, it was desired to set the bias current to be about equal in magnitude to the electron thermal current, as described in Fig. 2. This requires knowledge of the plasma density, whose average was well studied (Theis et al. 1984). However, the density at any altitude and solar-Venus angle was shown to vary by as much as a factor of 10 depending on solar wind conditions (Theis et al. 1984). Rather than setting the bias current according to the average density, it was decided to vary the bias current by a factor of about three around the average value in seven second steps, in hope of achieving the correct bias at least some of the time. The resulting VGA-7 bias current as a function of time is illustrated in Fig. (3a) with the negative values at times in sunlight before entering the penumbra or after exiting it. The measured spacecraft potential, in panel (3b) varied over a wide range with the large negative values near the beginning and end of the crossing signifying that the guessed plasma density was too small and the large peaks at other times occurred because the guessed density was too large. For a few minutes near the center of the crossing, the spacecraft potential was small and positive, which is the requirement found from the study of Fig. 2 for a good electric field measurement. Panel (3c) gives the measured electric field which had a spike every seven seconds due to the change of bias current and which showed unrealistically large values when the spacecraft potential did not have the required small positive value.

**Fig. 4:**
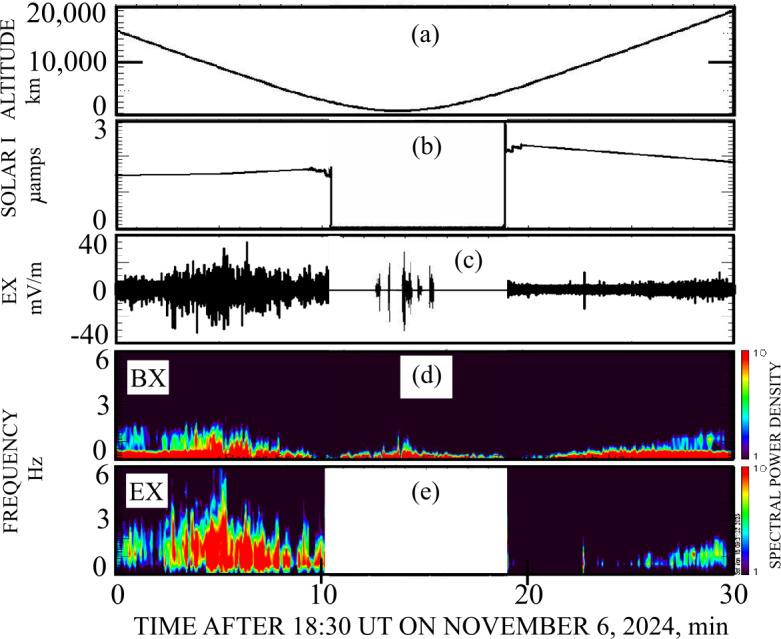
Panel (4a) gives the spacecraft altitude above Venus, which was as small as 400 km. The approximately eight minute interval during which the spacecraft was in the Venus shadow is defined by the loss of solar panel current in panel (4b). One component of the electric field is given in panel (4c), with the field in shadow appearing only when the spacecraft potential had the appropriate small positive value. Figures (4d) and (4e) give spectra of the magnetic field and electric field, with that for the electric field in shadow not illustrated because of the difficulty in obtaining good spectra over such short time intervals

Figure 4 presents 30 minutes of data surrounding the closest approach to Venus, which is illustrated in the spacecraft altitude plot in panel (4a). The approximately eight minute interval during which the spacecraft was in the Venus shadow is defined by the loss of solar panel current in panel (4b). One component of the electric field is given in panel (4c), with the field in shadow appearing only when the spacecraft potential of Fig. (3b) had the appropriate small positive value. In sunlight, the entire electric field is shown because the instrument was properly biased for sunlight conditions and good wave measurements were made (George et al. 2024). Figures (4d) and (4e) give the spectra as a function of time of the magnetic field and electric field, with that for the electric field in shadow not illustrated because of the difficulty in obtainIng good spectra over such short time intervals. It is noted that the electric field amplitude in shadow was similar to that in sunlight, panel (4c), and that there was significantly less magnetic field power in shadow than in sunlight, panel (4d). This suggests the possible absence of low frequency electromagnetic turbulence in the nightside magnetosphere.

**Fig. 5:**
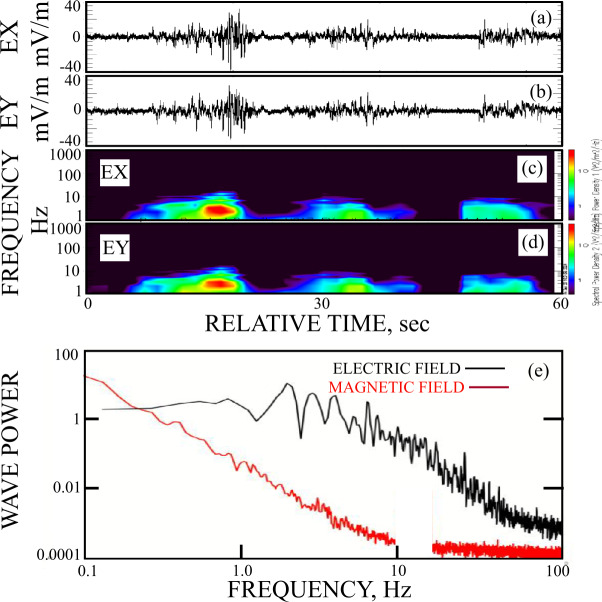
Panels (5a) and (5b) give the good electric field measurements in shadow as continuous plots. Their spectra, plotted in panels (5c) and (5d), show the presence of a few Hz electric field wave with no other features at frequencies below 1000 Hz. The average spectra of the electric and magnetic field in panel (5e) shows that no magnetic field signal was observed below 1000 Hz

In Figures (5a) and (5b) the good electric field measurements in shadow are given in single continuous plots. Their spectra, plotted in Figures (5c) and (5d), show the presence of a few Hz electric field wave with no other features at frequencies below 1000 Hz. The average spectrum of the electric field in panel (5e) confirms this result while the average spectrum of the magnetic field in panel (5e) shows that no magnetic field signal was observed below 1000 Hz other than 1/f noise (the magnetic field data near 10 Hz was removed because of magnetic noise from other sources at that frequency).

## Discussion

Good electric field measurements were made in the nightside Venus magnetosphere for the first time. During their one minute observation interval, the only wave mode observed was the few Hz electrostatic noise. This result, plus the magnetic field measurements, showed that there was little if any electromagnetic cascade turbulence, whistlers, or other electromagnetic waves below 1000 Hz. To the extent that this first one minute of good electric field measurements made in the nightside magnetosphere can be generalized, the plasma environment of Venus may be different from that of the terrestrial magnetosphere or the solar wind and deserves further field and particle measurements to understand the interaction between the solar wind and this unmagnetized body.

## Data Availability

The data used in this publication is available at spdf.gsfc.nasa.gov, at /data/spp/data/sci/fields/staging/l1b/dfb_wf_vdcX/2024, where X is 1, or 2, or 3, or 4 for each of the four antennas.

## Appendix: An Algorithm for Biasing Electric Field Antennas in the Sun’s Shadow

In practise, the floating potential of Fig. 2 is the potential of the spacecraft body and the biaed potential is the potential of an antenna. The difference between these two potentials is a quantity measured on the spacecraft. From Fig. 2 it is seen that this potential difference should be positive by an amount that is less than that for which the biased potential is positive. This maximum desired potential difference depends on the electron spectrum but is generally less than a few volts. Thus, a reasonable algorithm for biasing the antennas in shadow would be to modify the bias current until this potential is equal to V volts, where V is a constant that depends on the electron spectrum, is less than a few volts, and is determined by trial and error during the initial operation of the instrument.
